# Do age and performance status matter? A systematic review and network meta-analysis of immunotherapy studies in untreated advanced/metastatic non-oncogene addicted NSCLC

**DOI:** 10.3389/fimmu.2025.1635056

**Published:** 2025-09-23

**Authors:** Maria Anna Siciliano, Maria d’Apolito, Teresa Del Giudice, Giulio Caridà, Francesco Grillone, Giampiero Porzio, Raffaele Giusti, Pierfrancesco Tassone, Vito Barbieri, Pierosandro Tagliaferri

**Affiliations:** ^1^ Oncology Unit, Oncology Department, “Azienda Ospedaliero Universitaria (AOU) Dulbecco” Hospital, Catanzaro, Italy; ^2^ Oncology Unit, Tiberio Evoli Hospital, Melito di Porto Salvo, Reggio Calabria, Italy; ^3^ Department of Experimental and Clinical Medicine, Magna Græcia University, Catanzaro, Italy; ^4^ Tuscany Tumor Association, Oncological Home Care Service, Florence, Italy; ^5^ Oncology Unit, Azienda Ospedaliera-Universitaria Sant’Andrea, Rome, Italy

**Keywords:** non-small cell lung cancer, checkpoint inhibitors, network meta-analysis, systematic review, frail, older

## Abstract

**Background:**

Immune checkpoint inhibitors (ICIs) redefined the treatment of non-small cell lung cancer (NSCLC) but their efficacy in elderly and frail patients remains unclear due to immune-senescence and the underrepresentation of these populations in clinical trials. This systematic review and meta-analysis aimed to evaluate and rank first-line ICI-based therapies in NSCLC, stratified by age and performance status (PS).

**Methods:**

A comprehensive search for randomized controlled trials (RCTs) of ICI regimens, pairwise and network meta-analyses (NMA) based on age (<65, ≥65, ≥75 years) and PS (0 vs. 1) were conducted. Endpoints were overall survival (OS) and progression-free survival (PFS).

**Results:**

ICIs significantly improved OS and PFS versus chemotherapy (CT) in most subgroups. No OS benefit was observed in patients over 75 years. In younger patients, ICI+CT combinations (e.g. pembrolizumab+CT, cemiplimab+CT, camrelizumab+CT) ranked highest for OS and PFS. Among ≥65y patients, cemiplimab ranked first reaching statistical significance in most comparisons, while pembrolizumab was the most effective option for PFS. Stratified by PS, cemiplimab+CT ranked highest for OS in PS 0 patients, whereas cemiplimab was preferred in PS 1 patients. Overall, combination regimens were more effective in younger/fit patients, while monotherapy was more effective in older/PS 1 patients, suggesting a different benefit-risk balance. Anti-PD-1 therapies (alone or in combination) outperformed anti-PD-L1 and anti-CTLA-4 therapies in OS.

**Conclusions:**

This meta-analysis highlights how the efficacy of ICIs in advanced NSCLC varies by age and PS. These findings support a tailored approach to immunotherapy and emphasize the need for trials specifically targeting frail and elderly populations.

## 1 Introduction

The introduction of immune checkpoint inhibitors (ICIs) has paved the way for radical changes in the treatment of advanced/metastatic non-small cell lung cancer (NSCLC). The superiority of ICIs over standard chemotherapy (CT) has been widely demonstrated; however, there is now a rising need to identify which patients are most likely to benefit from immunotherapy (IT). In this challenging scenario, ICIs-based therapy in elderly/frail patients is still a relevant point of discussion that requires further investigation. In clinical practice, more than half of all patients with NSCLC are aged over 70 years, and nearly 10% are 80 years or older (1). Due to immune-senescence, there is a hypothetical risk of reduced efficacy and increased toxicity with ICIs. However, some data from clinical trials suggest that older patients might benefit from IT similarly to younger patients, with an acceptable safety profile (2). However, clinical trials mostly include patients with a performance status (PS) of 0–1 and median age at trial enrollment was about 10 years younger than the median age of NSCLC diagnosis. For this reason, data on ≥75 years or those with PS 2 patients are mostly derived from *post-hoc* analyses of small subgroups with limited statistical power and high risk of selection bias. Unlike CT, ICIs treatment is often given until disease progression or unacceptable toxicity, and the impact of this long-term treatment remains unclear. Additionally, the combination of IT and CT has become the standard of care in first-line NSCLC improving efficacy as compared to CT alone but also leading to a higher rate of adverse events. Therefore, there is a rising medical need to identify the most appropriated treatment strategy for frail and elderly populations to avoid over- or under-treatment and preventing useless toxicity. Indeed, this requires the design of pragmatic clinical trials that enroll populations as more similar to those observed in the real-life setting.

## 2 Methods

### 2.2 Systematic literature review

According to the Preferred Reporting Items for Systematic Reviews and Meta-analyses (PRISMA) guidelines, we conducted a systematic review using PubMed, Embase, Cochrane Library and relevant abstracts and presentations from major meeting databases (**Supplementary Figure S1**) (3). Timeframe was set from January 2010 to September 2024.

### 2.2 Data extraction and quality assessment

Data were independently extracted by two investigators (MAS and GC) performing the database searches and record selection, following a predefined protocol. Any disagreements were resolved through consensus. Both investigators assessed the risk of bias of the included studies using Cochrane risk of bias tool (4). The risk of bias was evaluated using the modified Cochrane Collaboration tool for randomized controlled trials (RCTs), evaluating the following domains: random sequence generation (selection bias), allocation concealment (selection bias), blinding of participants and investigators, blinding of outcome assessment (detection bias), incomplete outcome data (attrition bias), and selective reporting (reporting bias) (**Supplementary Figure S2**).

### 2.3 Study selection

Inclusion criteria: (1) phase 2 or 3 RCTs; (2) advanced/metastatic non-oncogene-addicted NSCLC; (3) comparison of two or more first-line treatments, including ICIs; (4) detailed outcomes including progression free survival (PFS) and overall survival (OS), stratified by age and/or PS. Studies that did not meet these criteria were excluded from the meta-analysis. Trials focusing on targeted therapy, radiotherapy, immune cells or cytokines, maintenance strategies or health-related quality of life were also excluded.

### 2.4 Endpoints

The primary endpoints of the meta-analysis were OS and PFS, analyzed in the overall population and stratified by age and PS. Specifically, subgroup analyses were conducted based on age (<65 years, ≥ 65 years, ≥75 years) and PS (0, ≥1). For both OS and PFS, hazard ratios (HRs) and corresponding confidence intervals (CIs) were extracted.

### 2.5 Pairwise meta-analysis

Pairwise meta-analyses were performed to compare IT-based therapy versus CT using Review Manager version 5.4 (Cochrane). For each pairwise meta-analysis, Cochrane’s Q test was used to assess statistical significance, with significance defined as a p-value ≤ 0.05. The presence of publication bias was excluded by visual inspection of funnel plots.

### 2.6 Network meta-analysis

Due to the heterogeneity of therapeutic strategies and the lack of direct comparisons, a Bayesian Network Meta-Analysis (NMA) was conducted. This analysis was performed using STATA (StataCorp, version 17) with a graphical interface and the mvmeta package. A Bayesian NMA was carried out for each outcome of interest using a Markov Chain Monte Carlo simulation with up to 30,000 iterations. Trials missing specific outcome data (e.g., HR for OS) were excluded from the corresponding NMA. The outcomes are reported with corresponding 95% credible intervals (CrIs). To identify the most credible treatment in the outcome of interest, we ranked the treatments using the surface under the cumulative ranking curve (SUCRA), derived by using command *sucra*. The closer the SUCRA value is to 1, the more probable the treatment is to rank as the best for the outcome of interest.

## 3 Results

### 3.1 Systematic literature review and description of eligible trials

A total of 289 of 3939 reports were screened by title and abstract. Further 257 articles were excluded from the qualitative evaluation. Thirty-two were selected by full text screening and were finally included in this analysis, involving 19.461 patients and 23 treatment regimens (**Supplementary Figure S3**). Of these, only studies reporting the necessary outcome data were included in the subsequent analyses. In the pairwise meta-analysis, 24 articles were included for OS and 20 for PFS. In the NMA, 26 articles were included in the analysis for OS and 22 for PFS. The experimental arm featured 6 ICI-monotherapy regimens [Keynote(KN)-024 (5), KN-042 (6), CheckMate(CM)-026 (7), IMpower(IM)-110 (8), Empower-lung 1 (9), IM-132 (10), Javelin Lung-100 (11), Mystic trial (12)], 3 dual-ICI strategy [CM-227 part I (13), KN-598 (14), Neptune (15)], 12 ICI/CT-regimens [KN-189 (16), NCT01285609 (17), KN-407 (18), CameL (19), CameL-Sq (20), Choice-01 (21), Empower-lung 3 (22), Gemstone-302 (23), IM-130 (24), IM-131 (25), CM-227 part II (26),Poseidon part I (27), Rationale-304 (28), Rationale-307 (29), Astrum-004 (30), Nippon (31), Orient-11 (32), Orient-12 (33), Innovent (34)], and 2 dual ICI/CT combinations [CM-9LA (35), Poseidon part II (27), CCTBG34 (36)]. Among them, 7 RCTs included only squamous (SQ) histology, 4 RCTs included only NSQ histology while the remaining studies included mixed histology. Regarding PD-L1 expression, 4 trials enrolled only patients with PD-L1 >50%, 1 trial included only patients with PD-L1>25% and 4 trials only patients with PD-L1 >1%. All other studies included patients with mixed PD-L1 expression levels. Details of included trials were provided in **Table 1**.

**Table 1:** Characteristics of included trials.

RCT	Type of trial	Year	Histology	PD-L1	Treatment comparison		Randomization	N° patients	Median FU (mo)	N° of patients for age			N° of patients for PS			Outcome
					Arm 1	Arm 2				<65y	≥65 y	>75y	0	1	2	
Astrum-004	Phase 3	2024	SQ	any	serplu+ct	ct	2:1	537	16.9	310	227	NA	92	445	NA	PFS
CameL	Phase 3	2024	NSQ	any	camre+ct	ct	1:1	412	65.2	314	98	NA	84	328	NA	OS, PFS
CameL-Sq	Phase 3	2024	SQ	any	camre+ct	ct	1:1	389	53.5	234	155	NA	81	308	NA	OS, PFS
CCTG-BR34	Phase 2	2022	any	any	durva+treme+ct	durva+treme	1:1	301	16.6	155	146	NA	92	209	NA	OS, PFS
Choice-01	Phase 3	2023	any	any	toripa+ct	ct	2:1	465	16.2	280	185	NA	102	363	NA	OS, PFS
CM 9LA	Phase 3	2024	any	any	nivo+ipi+ct	ct	1:1	719	64.5	354	295	70	227	492	NA	OS, PFS
CM 026	Phase 3	2017	any	≥1%	nivo	ct	1:1	541	13.5	281	198	62	178	357	5	OS, PFS
CM227 part 1	Phase 3	2021	any	any	nivo+ipi	ct	1:1	1739	29.3	912	642	185	596	1131	7	OS
CM227 part 2	Phase 3	2023	any	any	nivo+ct	ct	1:1	755	19.5	410	274	71	239	510	4	OS
EmpowerLung 1	Phase 3	2024	any	≥50%	cemi	ct	1:1	710	60	390	320	NA	192	518	NA	OS, PFS
EmpowerLung 3	Phase 3	2023	Any	any	Cemi+ct	ct	2:1	466	28.4	278	188	NA	69	393	NA	OS, PFS
GEMSTONE	Phase 3	2023	any	any	Suge+ct	ct	2:1	479	25.4	293	186	NA	84	395	NA	OS, PFS
NCT01285609	Phase 3	2017	SQ	/	ipi+ct	ct	1:1	749	12.5	380	298	71	259	485	5	OS
IM 110	Phase 3	2021	any	≥50%	atezo	ct	1:1	205	31.3	102	80	23	74	132	NA	OS
IM130	Phase 3	2019	NSQ	Any	atezo+ct	ct	2:1	723	18.5	362	276	85	297	424	1	OS, PFS
IM 131	Phase 3	2020	SQ	any	atezo+ct	ct	1:1	678	26.8	306	293	77	219	458	NA	OS, PFS
IM 132	Phase 3	2020	NSQ	any	atezo+ct	ct	1:1	578	28.4	320	257	NA	240	336	NA	OS, PFS
Innovent	Phase 3	2020	NSQ	any	sinti+ct	ct	2:1	397	8.9	NA	NA	NA	110	287	NA	PFS
Javelin lung 100	Phase 3	2024	any	≥1%	avelumab	ct	1:1	892	48.8	198	169	NA	314	575	NA	OS, PFS
KN 024	Phase 3	2020	any	≥50%	Pembro	ct	1:1	305	59.9	164	141	45	108	197	1	OS, PFS
KN 042	Phase 3	2022	any	≥1%	Pembro	ct	1:1	1274	61.1	707	567	129	390	884	NA	OS
KN 189	Phase 3	2021	NSQ	Any	pembro+ct	ct	2:1	616	31.0	312	304	NA	266	346	1	OS, PFS
KN 407	Phase 3	2020	SQ	any	pembro+ct	ct	1:1	559	14.3	254	305	NA	163	396	NA	OS, PFS
KN 598	Phase 3	2020	any	≥50%	pembro+ipi	pembro	1:1	568	20.6	281	287	NA	205	363	NA	OS, PFS
Mystic trial	Phase 3	2020	any	≥25%	durva	ct	1:1	488	30.2	163	162	NA	127	196	1	OS
Neptune	Phase 3	2023	any	any	durva+treme	ct	1;1	823	32.9	436	387	NA	314	507	NA	OS
Nippon	Phase 3	2024	any	any	pembro+ct	nivo+ipi+ct	1:1	295	15.3	NA	NA	47	136	159	NA	OS, PFS
Orient-11	Phase 3	2021	NSQ	any	sinti+ct	ct	2:1	397	22.9	NA	NA	NA	108	289	NA	OS, PFS
Orient-12	Phase 3	2021	SQ	any	sinti+ct	ct	1:1	357	12.9	NA	NA	NA	52	305	NA	PFS
Poseidon part 1	Phase 3	2024	any	any	durva+treme+ct	ct	1:1	675	63.4	367	308	NA	229	446	NA	OS
Poseidon part 2	Phase 3	2024	any	any	durva+ct	ct	1:1	675	63.4	345	330	NA	228	447	NA	OS
Rationale 304	Phase 3	2021	NSQ	any	tisle+ct	ct	2:1	334	9.8	237	97	NA	78	256	/	PFS
Rationale 307	Phase 3	2021	SQ	any	tisle+ct	ct	1:1	360	8.6	166	75	NA	63	178	NA	PFS

The table summarizes key features of each trial included in the analysis. Missing data are indicated as not available (NA), reflecting information not reported in the original publications. RCT, randomized clinical trial; PD-L1, programmed death-ligand 1; PS, performance status; SQ, squamous; NSQ, non squamous; serplu, serplulimab; ct, chemotherapy; camre, camrelizumab; durva, durvalumab; treme, tremelimumab; toripa, toripalimab; nivo, nivolumab; ipi, ipilimumab; cemi, cemiplimab; suge, sugemalimab; atezo, atezolizumab; sinti, sintilimab; pembro, pembrolizumab; tisle, tislelizumab; OS, overall survival; PFS, progression-free survival.

### 3.2 Pairwise meta-analysis

**Figure 1:**
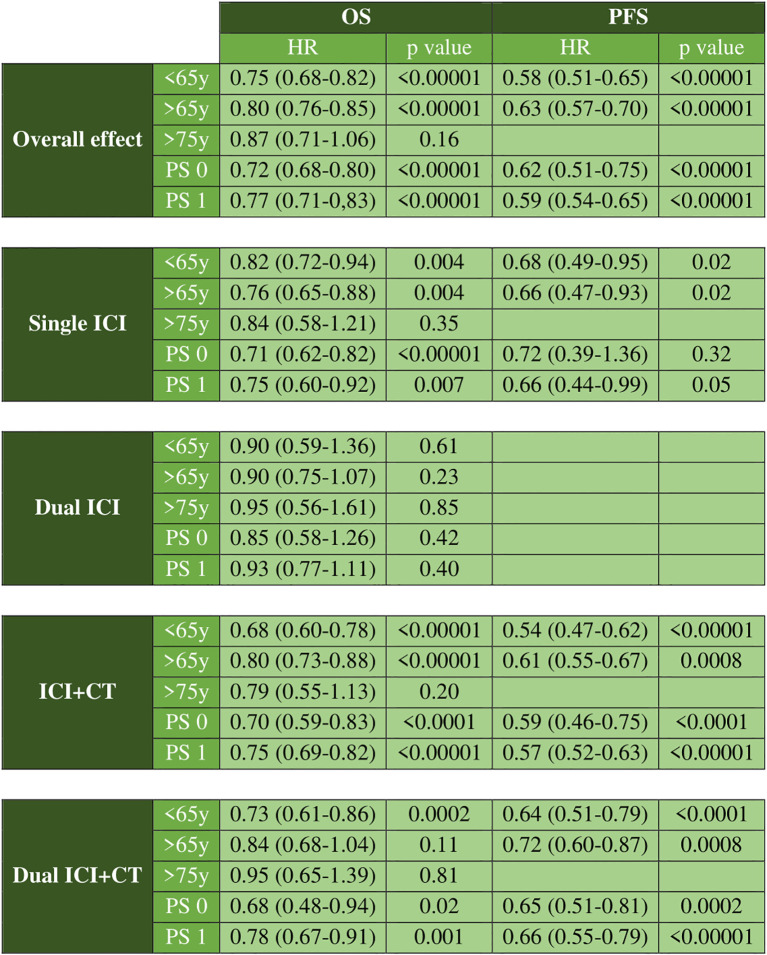
Graphical summary of pairwise meta-analysis results. Overall effect and results grouped by therapeutic regimen for OS and PFS.

To compare ICIs-based therapy with CT, a pairwise analysis stratified by patient age and PS was carried out. ICIs-based regimens were associated with a statistically significant reduction in the risk of death (<65 years: HR vs CT 0,75; 95% CI 0.68-0.82; ≥65 years: HR Vs CT 0.80; 95% CI 0.76-0.85; PS 0: HR Vs CT 0.87; 95% CI 0.68-0.80; PS 1: HR Vs CT 0.77; 95% CI 0.72-0.83) and disease progression (<65y: HR vs CT 0.58; 95% CI 0.51-0.65; ≥65y: HR Vs CT 0.63; 95% CI 0.57-0.70; PS 0: HR Vs CT 0.62; 95% CI 0.51-0.75; PS 1: HR Vs CT 0.59; 95% CI 0.54-0.65) (**Supplementary Figures S4**, **S5**). Across all subgroups, the impact of ICIs-based therapy on reducing the risk of disease progression was greater than its effect on OS. ICI monotherapy appeared to perform better in OS in patients with ≥65 years (HR 0.76; 95% CI 0.65-0.88), whereas in patients <65 years ICI plus CT were more effective. In younger patients, ICI/CT demonstrated a 58% reduction in the risk of death, compared to 16% for single ICI and 27% for dual ICI/CT. The combination of dual ICIs without CT did not improve OS compared to CT alone, although this finding is based on two studies only. Instead, for older patients, ICI monotherapy showed a slight advantage. In the <65 years subgroup, ICI/CT was also superior for PFS, as for OS (HR 0.54). Notably, in the ≥75 years population, ICI-based regimens were not associated with a statistically significant OS benefit compared to CT alone, though this data is based on few studies and patients (**Supplementary Figure S5**), resulting in low statistical power and limiting the certainty of this finding. PFS could not be analyzed in this subgroup due to insufficient data. Regarding PS, OS differences were minimal between groups, whereas for PFS, ICI/CT performed slightly better in both PS 0 and PS 1 patients (**Supplementary Figure S6**). Finally, breaking down the studies by ICIs type, anti-PD-1 therapy whether alone or in combination, performed better in OS than anti-PDL1 or anti-CTLA4 regimens (**Supplementary Figure S6**). A graphical summary of the pairwise meta-analysis results was shown in **Figure 1**.

### 3.3 NMA age analysis: OS and PFS

**Figure 2:**
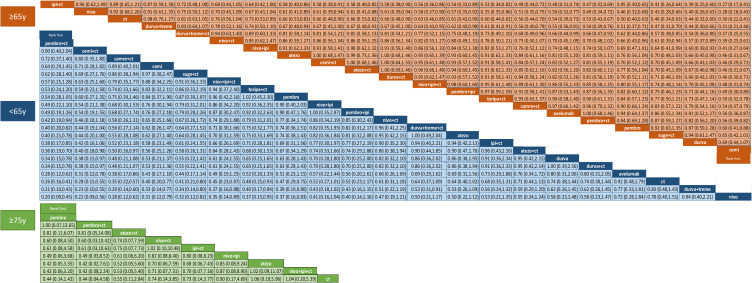
Hazard ratios and 95% CrI for OS of the NMA in age cohorts. The results are presented as column-defined treatment versus row-defined treatment.

**Figure 3:**
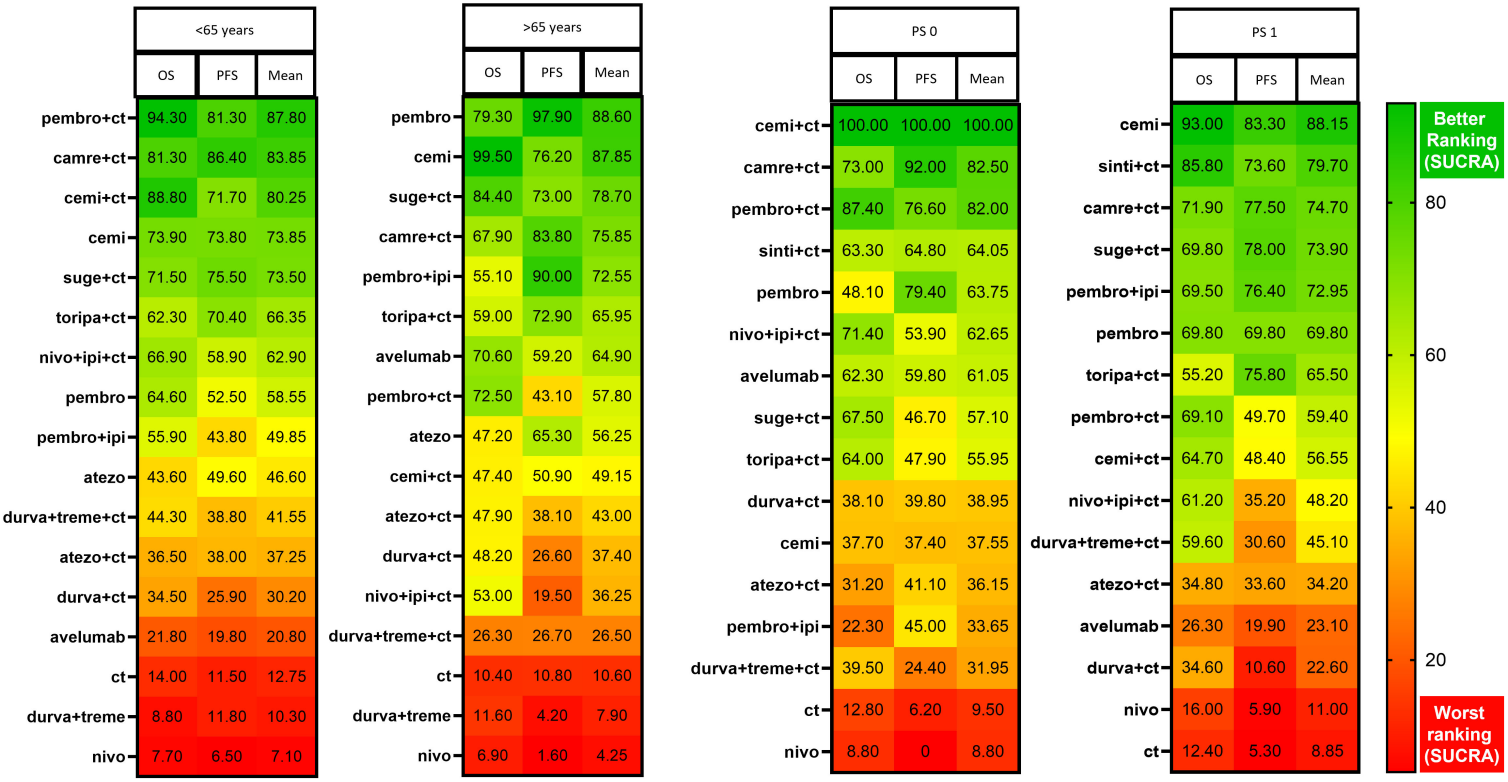
Ranking of treatments based on NMA across age and PS subgroups. All of the SUCRA values for each regimen with regard to PFS and OS. An average SUCRA and the average ranking are provided.

In the NMA analysis, pembrolizumab+CT (HR Vs CT 0.20; 95% CrI 0.09-0.45; SUCRA 94,1%), cemiplimab+CT (HR Vs CT 0.22; 95% CrI 0.09-0.56; SUCRA 89%) and camrelizumab+CT (HR Vs CT 0.28; 95% CrI 0.12-0.62; SUCRA 81,3%) ranked highest for OS in patients with <65 years. For patients ≥65 years, cemiplimab monotherapy ranked first in OS (HR 0.27; 95% CrI 0.17-0.41; SUCRA 99,5%) reaching statistical significance in most comparisons. Similar to the pairwise meta-analysis and facing the same statistical limitations, the NMA confirmed that in patients aged ≥75 years, ICIs-based therapy did not improve OS compared to CT (**Figure 2**; **Supplementary Figure S7**). For PFS analysis, camrelizumab+CT (HR 0.14; 95% CrI 0.05-0.38; SUCRA 86,4%) and pembrolizumab+CT (HR 0.15; 95% CrI 0.05-0.43; SUCRA 81.3%) ranked highest for patients <65 years. In contrast, pembrolizumab monotherapy had the highest probability of being the best treatment for reducing the risk of progression in patients ≥65 years (HR 0.14; 95% CrI 0.10-0.19; SUCRA 90%) (**Supplementary Figure S8**). Treatments with available data for both OS and PFS were compared using SUCRA rankings, visualized in a grouped heatmap and sorted by mean SUCRA value. Notably, in patients with <65 years, ICI/CT combinations had the highest probability of being the most effective in reducing the risk of death, while in patients with ≥65 years, ICI monotherapy ranked first (**Figure 3**).

### 3.4 NMA PS analysis: OS and PFS

**Figure 4:**
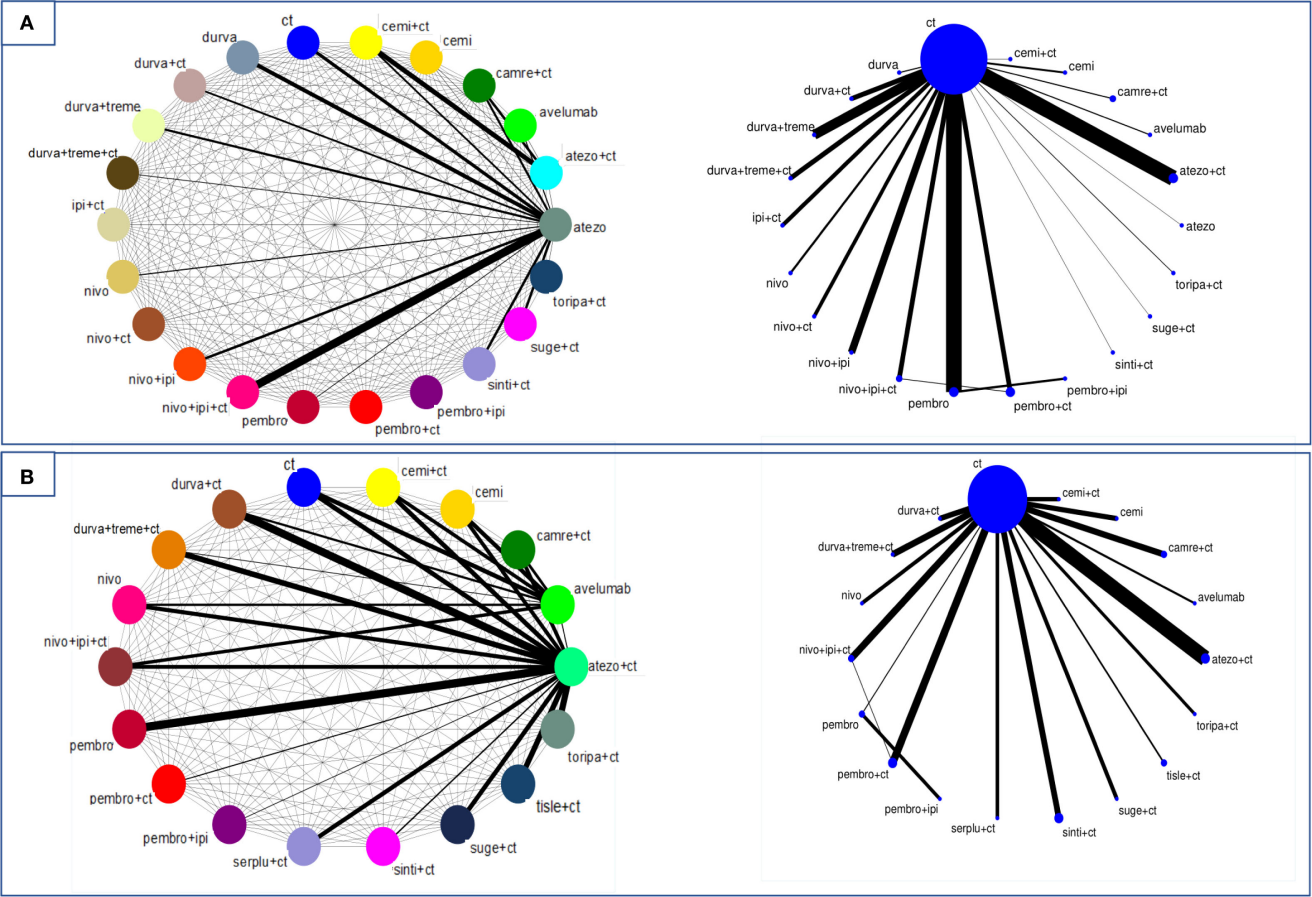
Network plot of direct (right) and indirect (left) comparison of the studies included in the analysis for OS **(A)** and PFS **(B)** in the PS cohort. Each circular node represents a treatment type. The thickness of the lines is proportional to the number of patients in head-to-head comparisons.

In terms of reducing the risk of death in patients with PS 0, cemiplimab/CT ranked first in NMA with a statistically significant advantage over all other treatments (HR Vs CT 0.05; 95% CrI 0.02-0.12; SUCRA 100%). In contrast, pembrolizumab/CT ranked second (HR Vs CT 0.22; 95% CrI 0.10-0.49; SUCRA 87,4%) (**Figure 4**; **Supplementary Figure S9**). Instead, for patients with PS 1, cemiplimab ranked first in OS. However, both pembrolizumab/CT and cemiplimab/CT performed worse in PS 1 patients compared to those with PS 0. Conversely, pembrolizumab monotherapy ranked higher in the PS 0 subgroup. For PFS, camrelizumab/CT and cemiplimab/CT ranked first. Notably, all ICIs-based treatments outperformed CT (**Figure 4**; **Supplementary Figure S9**). A grouped heatmap for PS is shown in **Figure 3**, highlighting that in PS 0 patients ICI/CT combinations had the highest probability of being the best treatment for both OS and PFS. Meanwhile, in PS 1 patients, cemiplimab monotherapy ranked first.

## 4 Discussion

IT with ICIs has become a cornerstone in the treatment of advanced/metastatic NSCLC. There is growing interest in defining the efficacy and safety of this approach in older and frail patients, identifying the most effective treatments in this setting. Due to the poorly characterized immune-senescence phenomenon, it remains unclear whether ICIs-based treatments are less effective in older patients. Aging-related changes in the immune system, collectively known as immune-senescence, may contribute to resistance to IT (37). Thymic involution and chronic antigenic stimulation cause naive T cells to convert into virtual memory cells, potentially impairing immune responses to pathogens and tumors. Additionally, the reduced number of naive CD8+ T cells, along with an increase in antigen-experienced CD4+ and CD8+ T cells, leads to a diminished capacity to respond to newly encountered antigens. Furthermore, decrease of costimulatory signals (like CD28 and CD27 on T cell) or the upregulation of Tim-3 and CD57 were described in the elderly and have been linked to reduced response to ICIs (38). Immune-senescence phenomenon involves also B cells and innate immune response (39).

Despite this, individual clinical trials have shown a similar survival benefit of ICIs compared to CT across younger and older patients with an acceptable safety profile, based however on underpowered *post-hoc* analyses unable to definitely address this crucial point. At this aim, this systematic review and meta-analysis were carried out to summarize and rank the efficacy of first line ICIs-based treatments in advanced/metastatic NSCLC, considering age and PS. Our findings confirmed that ICIs-based regimens significantly improved OS and PFS compared to CT in all subgroups, with a greater impact on PFS. A previous meta-analysis by Zhou et al. found that PFS benefits were more pronounced than OS benefit in the first-line setting, whereas the opposite was observed in later treatments lines (40). For patients over 75 years, our analysis did not demonstrate a statistically significant OS benefit of ICIs over CT. This finding may be due to limited data and small sample size of this subgroup, thereby limiting the confidence in the observed outcome. PFS could not be analyzed due to insufficient data (only 3 studies). Notably, only IM-131 (25) reported a statistical significant PFS (77 patients), whereas CM-9LA (70 patients) and NIPPON (47 patients) showed negative outcomes (31, 35). Caution is required in interpreting these results, and validation in larger cohorts of elderly patients is needed.

In older patients, ICI monotherapy seems to perform better in the pairwise as well as NMA, while ICI+CT was the best strategy in younger patients. In patients aged over 65 years ICI+CT (pembrolizumab+CT, cemiplimab+CT and camrelizumab+CT) ranked highest in OS, though without statistical significance in all comparisons. In patients over 65 years cemiplimab monotherapy ranked first in OS with statistical significance for most comparisons. For PFS, camrelizumab+CT and pembrolizumab+CT ranked highest in <65 years (with a statistical significance only over nivolumab, CT, durvalumab+tremelimumab, avelumab, and atezolizumab+CT) whereas pembrolizumab monotherapy had the highest probability of reducing disease progression in ≥65 years (non-statistically significant comparisons: pembrolizumab+ipilimumab, camrelizumab+CT, cemiplimab, toripalimab+CT, serplulimab+CT). When stratified by PS, OS differences between PS 0 and PS 1 were minimal whereas for PFS ICI/CT performed slightly better in both PS 0 and PS 1. In PS 0 patients, cemiplimab+CT ranked first in OS (statistically significant vs. all treatments) with pembrolizumab+CT ranked second. In PS 1, cemiplimab monotherapy performed best in OS, while cemiplimab+CT and pembrolizumab+CT performed worse than in PS 0.

These rankings led us to conclude that: i) combination therapies appear more effective in younger and fit patients, potentially due to a stronger immune response and/or better treatment tolerance; ii) mono immune-therapy appeared more effective in older and PS 1 patients, likely reflecting a different benefit-risk balance and reduced treatment tolerance. Furthermore, our analysis of ICIs type demonstrated that anti-PD1 therapy (alone or in combination), outperformed anti-PD-L1 and anti-CTLA-4 regimens in OS.

Unlike previous meta-analyses investigating this field, our study provides a more comprehensive comparison of available treatment strategies in this setting, including the largest number of RCTs and the most recent data updates. Moreover, at the best of our knowledge this is the first NMA considering both age and PS to investigate the frailty scenario.

Our results should be evaluated in the current landscape of studies focused on the relevance of age and PS. Landre et al. performed a NMA considering only patients over 65 years, demonstrating OS and PFS benefit of the anti-PD-1/PD-L1 plus CT, but with no consistent evidence in patients aged over 75 years (41). In a NMA focused only on older patients with PDL-1 ≥50%, cemiplimab monotherapy emerged as the preferred treatment strategy, consistent with our previous findings (42). Instead, Sun et al. found similar OS efficacy across age groups but no PFS benefit in either young and older patients (though their analysis included only 8 trials) (43). Regarding PS, an interesting meta-analysis in real-world advanced NSCLC patients with PS ≥2 reported detrimental effects of ICIs therapy on OS, PFS and ORR, raising concerns about treatment suitability in frail patients (42). All together these studies support our finding of relevant role of age and PS in predicting effectiveness of immune-oncology strategies. However, it should be emphasized the under-representation of frail patients in clinical trials, limiting the applicability of trials data in real-world clinical practice. Chronological age and PS alone do not fully capture a patient’s condition; a comprehensive, multidisciplinary evaluation is necessary to assess overall PS and health. PS deterioration may result from various factors, including disease burden, comorbidities, age, and the overall frailty, making it critical to differentiate between cancer-related PS decline and other underlying conditions. Comprehensive Geriatric Assessment (CGA) represents a well-established, multidimensional approach for evaluating older patients. Initially developed within geriatrics, CGA has been increasingly applied in oncology to guide treatment decisions in elderly cancer patients and to better characterize frailty (44). Recent prospective data in elderly NSCLC patients undergoing CGA suggest that frailty, comorbidities, and low albumin levels are associated with worse survival outcomes and higher toxicity, highlighting the importance of CGA-guided treatment decisions (45). Among elderly patients, safety concerns remain a key consideration due to impaired renal and/or cardiac function, increased comorbidities, declining organ function and cognitive impairment (46). Studies suggest that ICIs safety profile is comparable between older/frail and fit patients, though CT-based combinations inevitably increase toxicity (47–49). Therefore, adequate geriatric screening is essential to prevent both over- or under-treatment in this population.

Several limitations of this meta-analysis should be acknowledged. Firstly, data were extrapolated from published RCTs rather than individual patient data. Heterogeneity was evident when pooling data across different ICIs or CT backbone, trial design, histology and PD-L1 expression. Formal analyses of safety and toxicity stratified by age/PS could not be performed because data were not reported in clinical trials. Additionally, several data points relied on *post-hoc* analyses and ongoing trials have yet to report survival outcomes, introducing potential bias. Longer follow-up is needed to fully assess the long-term impact of ICIs on OS. Furthermore, in older patients (≥75 years), non-cancer-related mortality may significantly interfere with OS outcomes in phase III trials.

## 5 Conclusions

This systematic review and meta-analysis confirmed that ICI-based therapy significantly improved OS and PFS compared to CT across all subgroups, except for patients with ≥75 years. The best treatment strategy seems to vary by age and PS, with ICI monotherapy being most effective in older/PS 1 patients, while ICI+CT combinations performed better in younger/PS 0 patients. In conclusion, there is an urgent need to design future RCTs focusing on the use of IT in frail populations as a whole, improving patients stratification using geriatric tools. Considering that the rate of end-of-life IT is increasing, this could guide the clinicians in discriminating situations of over- or under-treatment, providing recommendations for clinical practice in selecting optimal strategies in these patients.

## Data Availability

The original contributions presented in the study are included in the article/**Supplementary Material**. Further inquiries can be directed to the corresponding author.
