# Supplementary material for: Do age and performance status matter? A systematic review and network meta-analysis of immunotherapy studies in untreated advanced/metastatic non-oncogene addicted NSCLC
Source: Front Immunol. 2025 Sep 23;16:1635056. doi: 10.3389/fimmu.2025.1635056 (PMC12500590; doi:10.3389/fimmu.2025.1635056)
Supplement: Supplementary file 1 [file DataSheet1.docx]

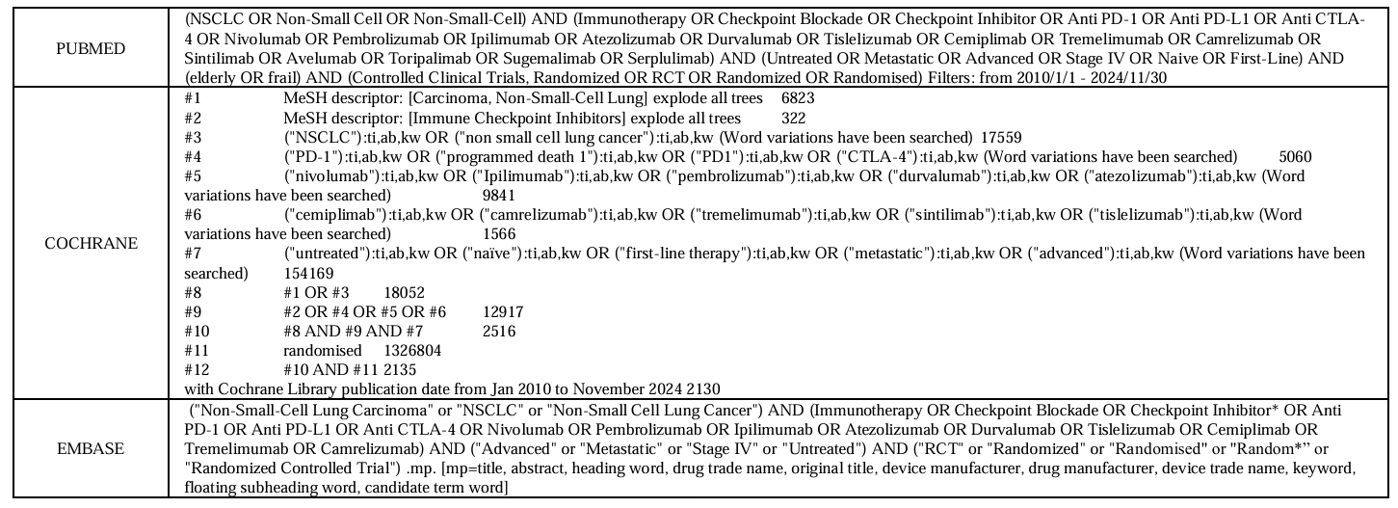


**Figure S1.** **Search strategy.** NSCLC, non-small cell lung cancer; PD-1 programmed death protein-1; PD-L1, programmed death-ligand 1, CTLA-4, Cytotoxic T-Lymphocyte Antigen 4); RCT, randomized controlled trial.


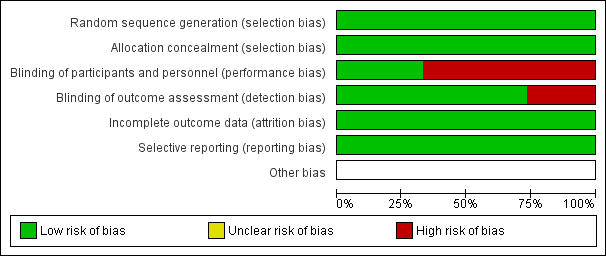

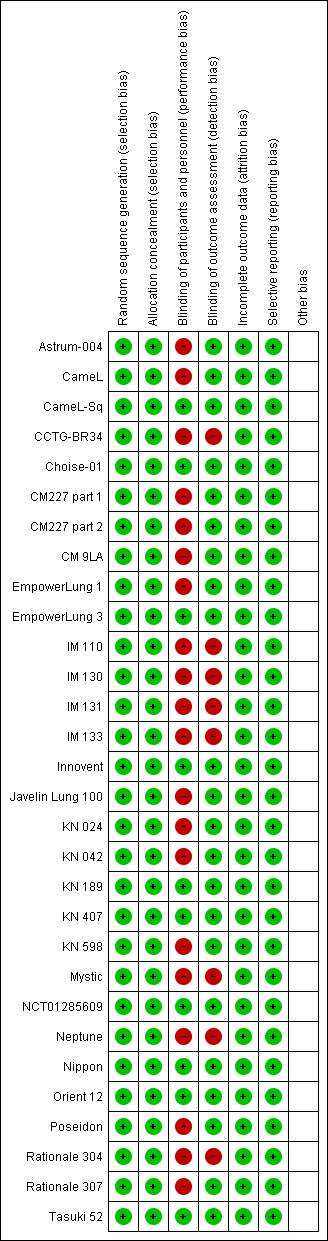


**Figure S2. Risk of bias assessment.** Risk of bias assessment for each included study.


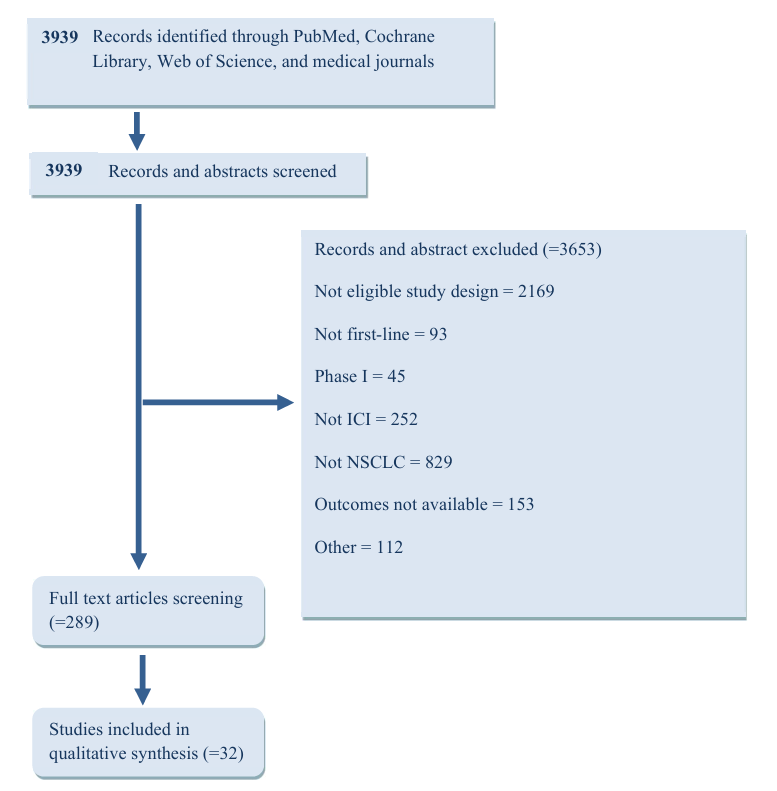


**Figure S3. Preferred Reporting Items for Systematic Reviews and Meta-Analyses (PRISMA) flow chart diagram.** RCT, randomized clinical trial; ICI, immune checkpoint inhibitor; NSCLC, non small cell lung cancer.


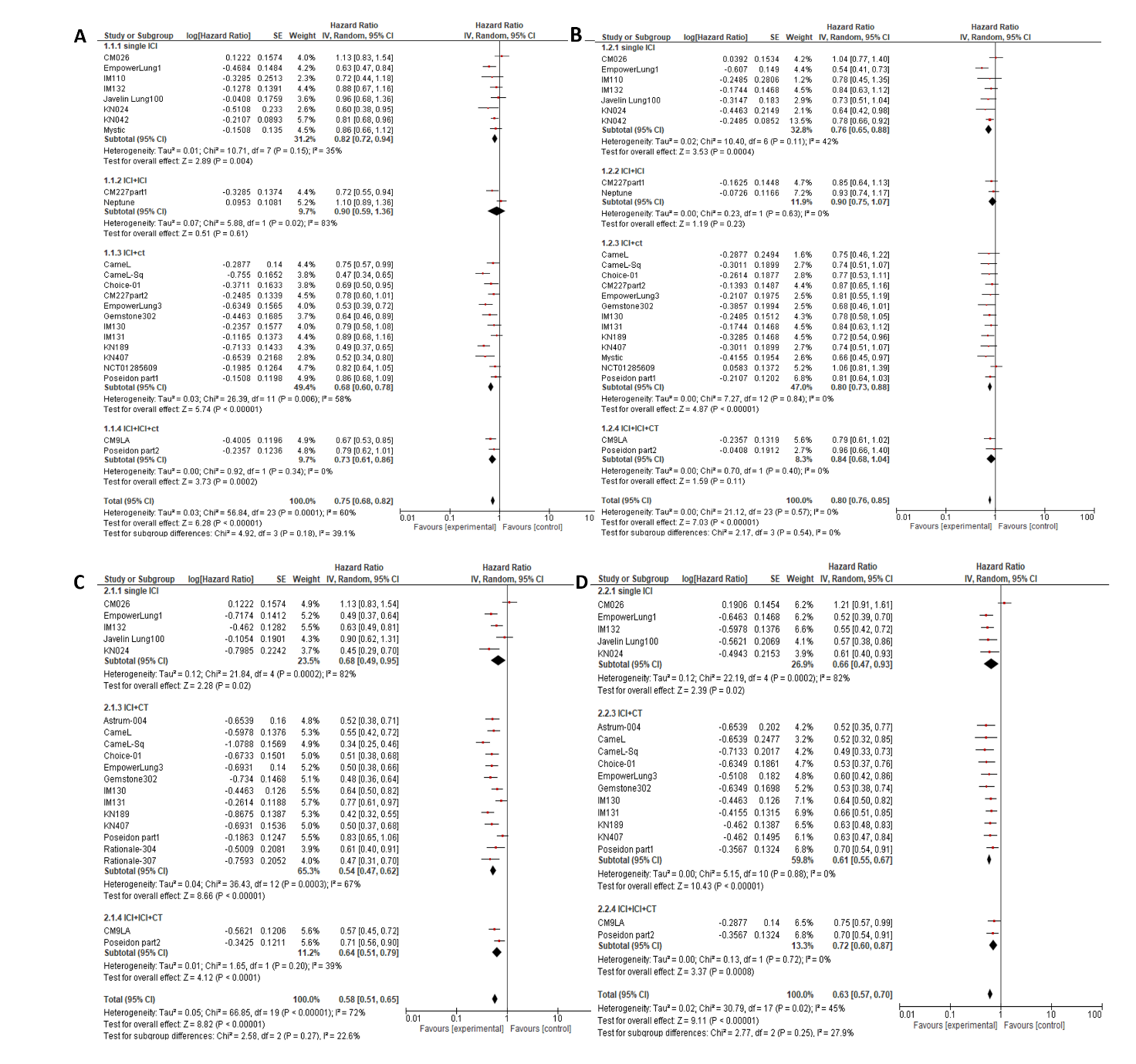


**Figure S4. Pooled HR for OS and PFS on head-to-head comparison for <65years (A;C) and ≥65 years (B;D) patients.** Immune checkpoint inhibitor-based regimens represent the experimental group. Subgroups have been created according to the type of therapeutic regimen.


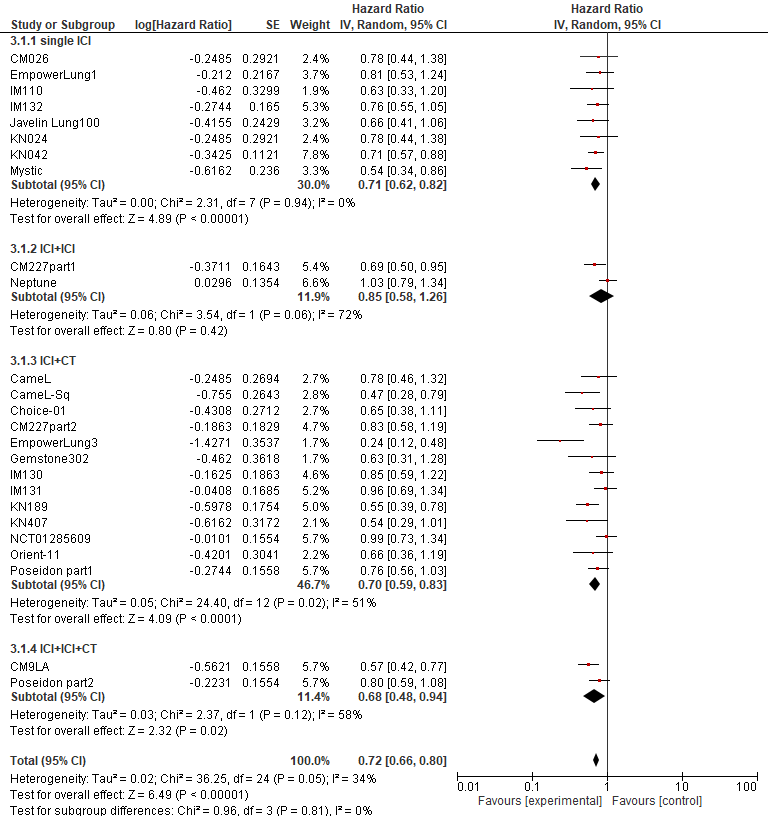

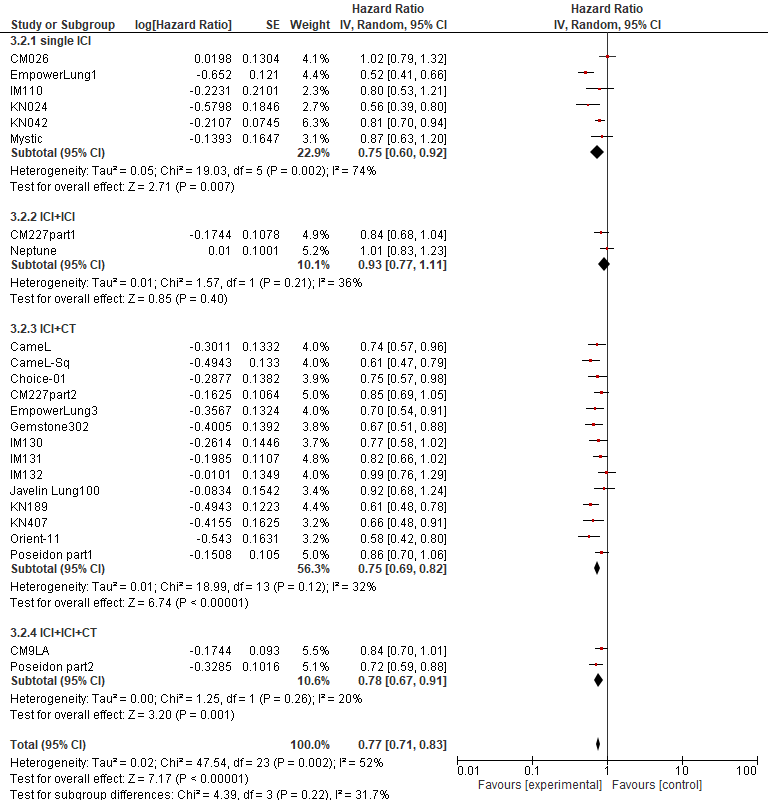

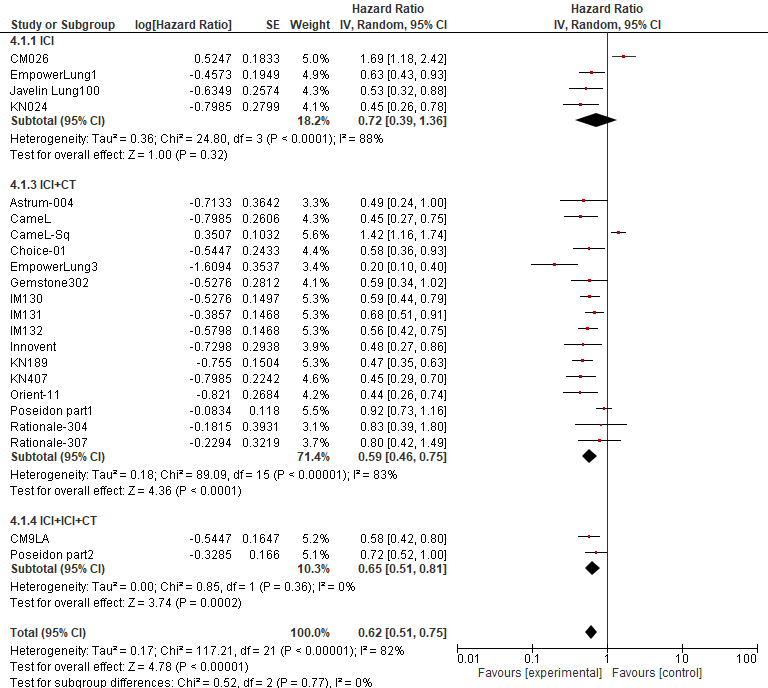

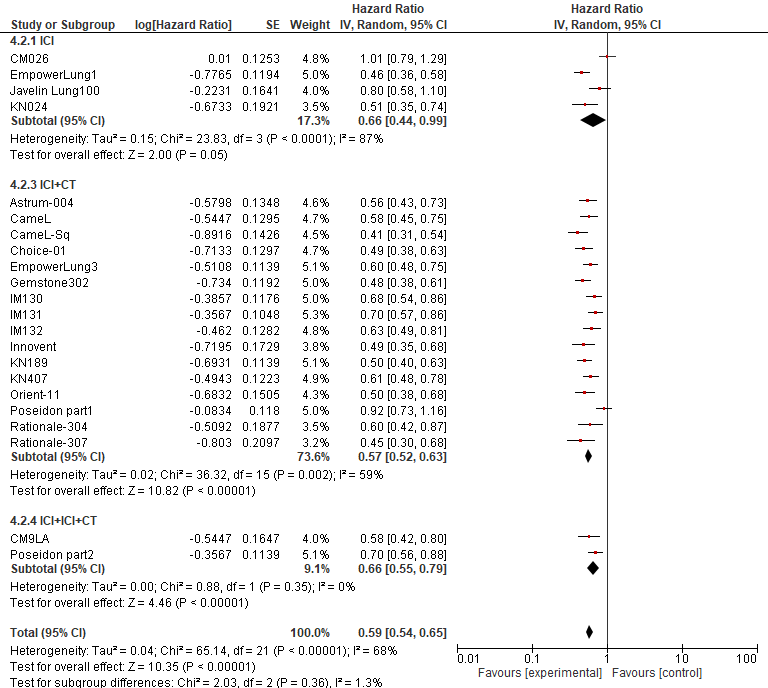


A


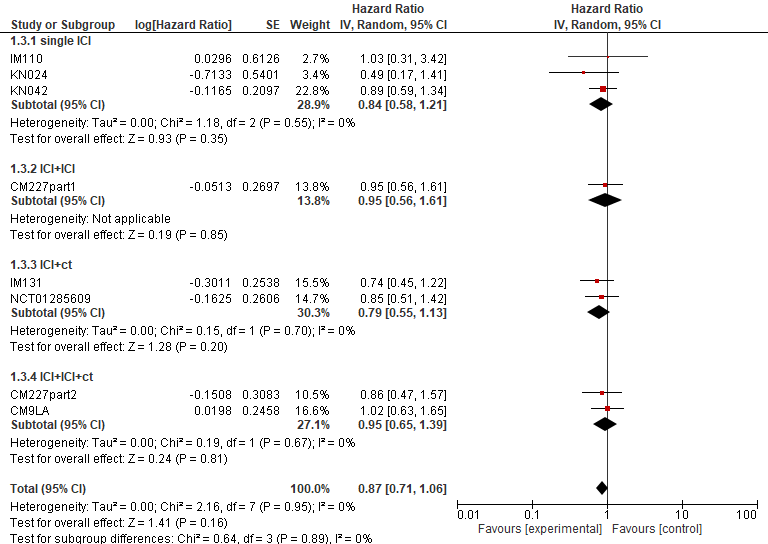


B

**Figure S5**. **Pooled HR for OS (A) and PFS (B) on head-to-head comparison for PS 0 (left) and PS 1 (right) patients.** Immune checkpoint inhibitor-based regimens represent the experimental group. Subgroups have been created according to the type of therapeutic regimen.

**
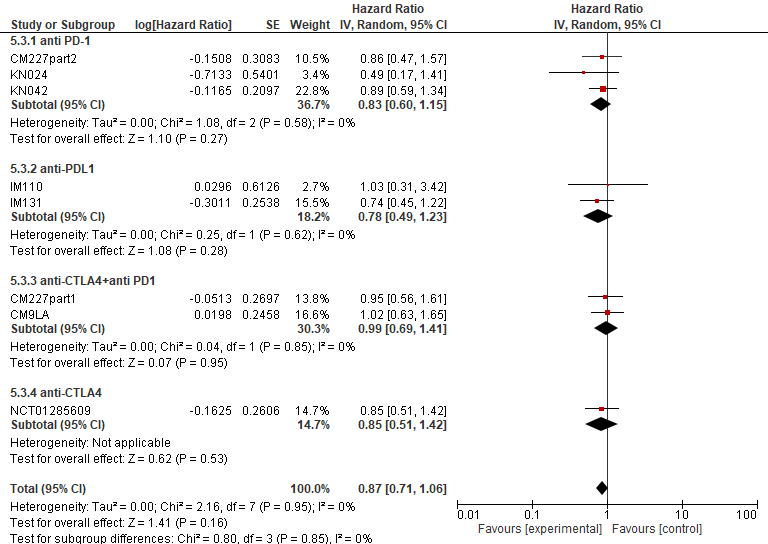
**

**Figure S6.** **Pooled HR for OS on head-to-head comparison for ≥75 years patients.**

Immune checkpoint inhibitor-based regimen represents the experimental group. Subgroups have been created according to the therapeutic regimen.

**A**

**B**


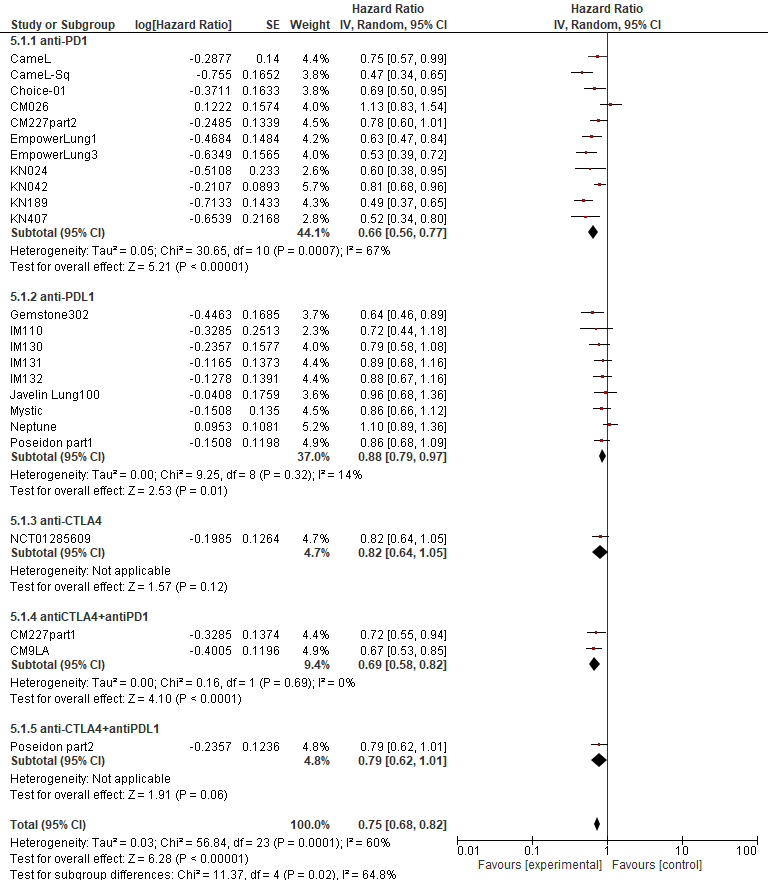

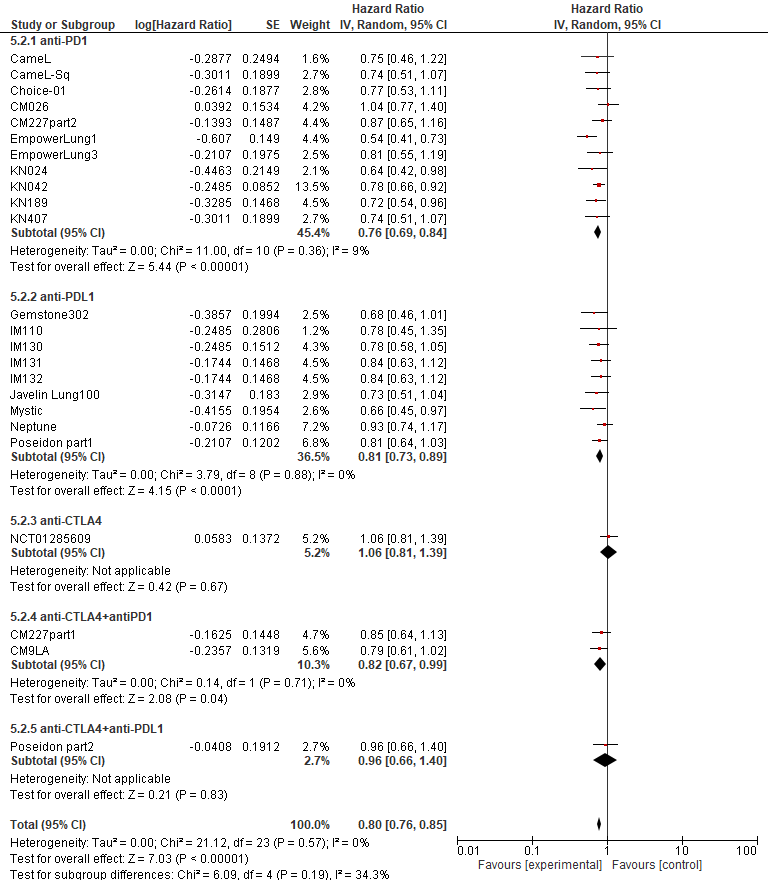

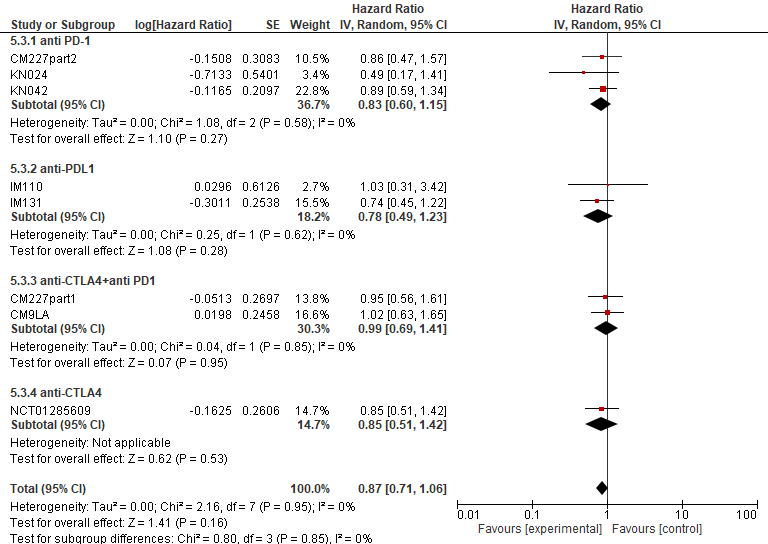


**C**

**Figure S7.** **Pooled HR for OS on head-to-head comparison for <65years (A), ≥65 years (B) and ≥75years (C) patients grouped by ICI-type.** Immune checkpoint inhibitor-based regimens represent the experimental group. Subgroups have been created according to the type of ICIs.

**
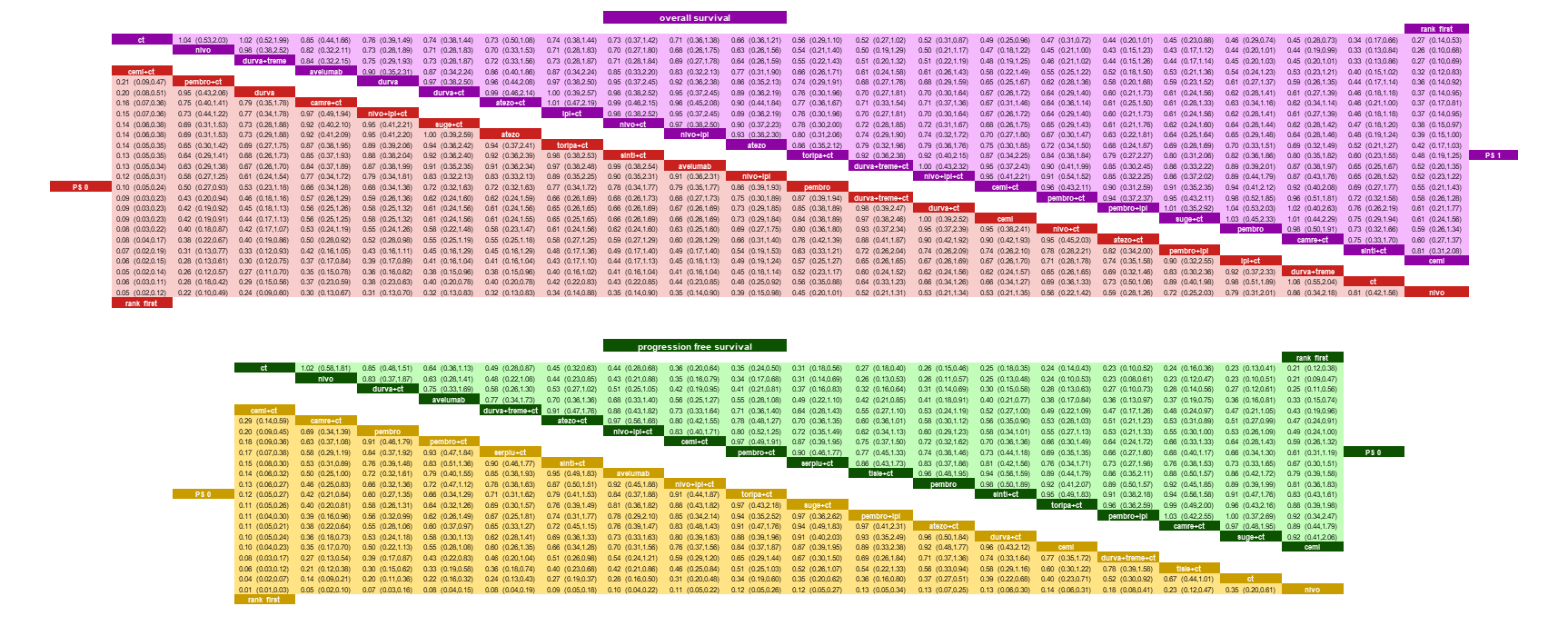
Figure S8. Hazard ratios and 95% CrI and network-plot structure for PFS of the NMA for all included trials in age cohorts.** The results are presented as column-defined treatment versus row-defined treatment.

**Figure S9. Hazard ratios and 95% CrI for OS and PFS of the NMA and network-plot structure for all included trials in PS cohorts.** The results are presented as column-defined treatment versus row-defined treatment.
